# Synergistic Antibacterial and Antibiofilm Effects of Clindamycin and Zinc Oxide Nanoparticles Against Pathogenic Oral *Bacillus* Species

**DOI:** 10.3390/pathogens14020138

**Published:** 2025-02-02

**Authors:** Maha A. Khalil, Tahany M. Alzaidi, Mohammed Hussein M. Alsharbaty, Sameh S. Ali, Michael Schagerl, Hesham M. Elhariry, Tamer A. Aboshady

**Affiliations:** 1 Department of Biology, College of Science, Taif University, Taif 21944, Saudi Arabia; maha.k@tu.edu.sa (M.A.K.); tahany@taif.edu.sa (T.M.A.); 2 Branch of Prosthodontics, College of Dentistry, University of Al-Ameed, Karbala 56001, Iraq; hussseinalsharbaty1986@gmail.com; 3 Botany and Microbiology Department, Faculty of Science, Tanta University, Tanta 31527, Egypt; 4 Department of Functional and Evolutionary Ecology, University of Vienna, Djerassiplatz 1, A-1030 Vienna, Austria; 5 Department of Food Science, Faculty of Agriculture, Ain Shams University, Cairo 11241, Egypt; helhariry@agr.asu.edu.eg; 6 Oral and Maxillofacial Surgery and Diagnostic Sciences, College of Dentistry, Taif University, Taif 21944, Saudi Arabia; t.almeer@tudent.edu.sa

**Keywords:** oral bacterial pathogens, drug resistance, metal oxide nanoparticles, cytotoxicity, biofilm-associated genes, antibacterial

## Abstract

Oral bacterial pathogens, including *Bacillus* species, form biofilms that enhance antibiotic resistance, promote bacterial adherence, and maintain structural integrity. The ability of bacteria to form biofilms is directly linked to several oral diseases, including gingivitis, dental caries, periodontitis, periapical periodontitis, and peri-implantitis. These biofilms act as a predisposing factor for such infections. Nanoparticles, known for their strong antibacterial properties, can target specific biofilm-forming microorganisms without disturbing the normal microflora of the oral cavity. This study focuses on the biofilm-forming ability and clindamycin (CM) resistance of *Bacillus* species found in the oral cavity. It aims to evaluate the antibacterial and antibiofilm properties of zinc oxide nanoparticles (ZnO-NPs) against oral *Bacillus* species and assess the effectiveness of combining CM with ZnO-NPs in reducing antibiotic resistance. The antibacterial susceptibility of *Bacillus* isolates was tested using ZnO-NPs and CM, demonstrating synergistic effects that reduced the minimum inhibitory concentrations by up to 8-fold. The fractional inhibitory concentration (FIC) index indicated a significant synergistic effect in most strains, with FIC values ranging from 0.375 to 0.5. It was found that the majority of *Bacillus* strains exhibited significant biofilm-forming capabilities, which were reduced when treated with the ZnO-NPs and CM combination. The study also evaluated the cytotoxicity of ZnO-NPs on cancer cells (CAL27) and normal fibroblasts (HFB4). CAL27 cells showed stronger cytotoxicity, with an IC_50_ of 52.15 µg/mL, compared to HFB4 cells, which had an IC_50_ of 36.3 µg/mL. Genetic analysis revealed the presence of biofilm-associated genes such as *sipW* and *tasA*, along with antibiotic resistance genes (*ermC*), which correlated with the observed biofilm phenotypes. Overall, this study demonstrates the potential of combining ZnO-NPs with CM to overcome antibiotic resistance and biofilm formation in the oral bacterial pathogens, *Bacillus* species. These findings suggest new approaches for developing more effective dental treatments targeting oral biofilm-associated infections and antibiotic resistance.

## 1. Introduction

Plaque, also referred to as calculus, is a biofilm composed of bacteria and other organisms that forms during the chewing of food. This biofilm is made up of a diverse array of microorganisms that are densely packed together and stabilized by a network of organic polymers produced by both bacterial activity and saliva [1,2,3]. Similar to biofilms found in various environments, dental plaque forms on the surfaces of teeth in the oral cavity. When bacteria aggregate and adhere to a surface, a biofilm is created. These bacteria synthesize an extracellular polymeric matrix that encapsulates them [4]. Research shows that this extracellular polysaccharide matrix is synthesized by bacterial species such as *Staphylococcus* sp., *Streptococcus* sp., *Bacillus* sp., *Enterobacter* sp., *Corynebacterium* sp., *Micrococcus* sp., and *Klebsiella* sp. [3,5]. *Bacillus* sp. is commonly present in the oral cavity. These species are typically considered transient in a healthy individual’s mouth. Initially nonpathogenic, they can become opportunistic and lead to various oral diseases [6,7].

Biofilm-forming bacteria differ from planktonic bacteria, which move freely in aquatic environments. Plaque biofilm bacteria can respond to various signals, including nutritional cues and cellular recognition of specific and non-specific surface attachment sites [8,9]. Biofilms can develop on a wide range of oral surfaces, such as enamel, dentin, gingiva, cementum, oral mucosa, carious lesions, restorations, dental implants, and dentures. The exposed root surface and coronal enamel surface are quickly colonized by plaque. Microbiota proliferate more rapidly on exposed root dentin surfaces than on enamel surfaces due to the irregular geometry of the former [10]. Surface-bound bacteria have a higher likelihood of survival and natural selection compared to their planktonic counterparts. Bacteria present in dental plaque exhibit greater resistance to antibiotic treatment than waterborne bacteria. The extracellular polysaccharides produced by plaque bacteria offer protection against various threats, including antibiotics, antibodies, surfactants, bacteriophages, and white blood cells [11]. Bacteria within a biofilm can also develop resistance to disinfectants. The minimum inhibitory concentration (MIC) of antimicrobial drugs is significantly higher (up to 1000-fold) for bacteria in biofilms compared to planktonic bacteria [12]. This condition is linked to tooth loss and poses a significant risk to global health [13,14].

In light of the growing prevalence of microorganisms that are resistant to conventional antibiotics, there is an immediate and pressing requirement for the development of additional effective treatments [15,16]. Clindamycin (CM) is an antibiotic that works by binding to the large subunit of the ribosome [17]. The mrsA and mefA genes encode efflux pump activity [18,19], while the linA gene is associated with O-nucleotidyltransferase activity [20], which is believed to inactivate CM. The prevention of plaque formation and maturation may be achievable through the discovery of new, strategic, and effective antibacterial agents [21,22]. Understanding the potential of metal nanoparticles to enhance the efficacy of antimicrobials against bacteria responsible for gum disease is crucial.

As a potential treatment method for conditions associated with biofilms, nanotechnology has demonstrated a great deal of promise. As vehicles for the administration of medication, nanoparticles offer a number of benefits, including a reduction in medication-related adverse effects and a prolonged release of the drug. The purported antibacterial properties of a number of nanoparticles have been the subject of a significant amount of research. Metallic oxide nanoparticles, such as zinc oxide nanoparticles (ZnO-NPs), exhibit low cytotoxicity and show significant potential as drug delivery systems. Research indicates that the antibacterial and antibiofilm properties of nanomaterials are strongly influenced by factors like their shape and surface charge [23,24,25,26]. ZnO-NPs provide several benefits, including improved solubility, bioavailability, and biocompatibility. They can interact with biomolecules, localize within organs, and contribute to cellular homeostasis [27]. ZnO-NPs also offer lower toxicity and cost compared to alternative nanomaterials, making them suitable for a wide range of applications, including cancer therapy, infection and inflammation prevention, and diabetes management [28,29]. Additionally, ZnO-NPs are characterized by their catalytic efficiency, chemical stability, and exceptional adsorption capability [30]. Therefore, this study aims to evaluate the antibacterial and antibiofilm properties of ZnO-NPs against oral *Bacillus* species and to assess the effectiveness of combining CM with ZnO-NPs in reducing antibiotic resistance.

## 2. Material and Methods

### 2.1. Metal Oxide Nanoparticles Used in This Study

The precipitation method described by Khalil et al. [31] was used to synthesize ZnO-NPs. Solutions A and B were prepared from zinc nitrate hexahydrate (Zn(NO_3_)_2_·6H_2_O) at a concentration of 0.1 M and sodium carbonate (Na_2_CO_3_) at a concentration of 0.12 M, respectively. Solution A was then added to solution B incrementally, drop by drop, while the mixture was stirred vigorously. The white precipitate was filtered and washed with distilled water three times after its formation. The ZnO-NPs were characterized using UV–Vis spectroscopy [32], transmission electron microscopy (TEM) [33], and energy dispersive X-ray analysis (EDX) [34]. The results revealed that the ZnO-NPs exhibited an absorption peak in the range of 360–380 nm, and the EDX analysis confirmed significant zinc signatures within the expected range. The particles were previously described by Khalil et al. [31] as spherical, well separated, and non-aggregated, with an average size of 7.6 ± 0.5 nm.

### 2.2. Sample Collection

Clinicians from the Medical Diagnosis and Infection Control Unit at Taif University’s Faculty of Dentistry collected oral cavity samples (n = 50) by swabbing the gingiva, subgingiva, and both the roof and floor of the buccal cavity to isolate pathogenic *Bacillus* species. The clinicians followed the guidelines and standard protocols in accordance with the Declaration of Helsinki [35]. The study was approved by the Research Ethics Committee of Taif University, with approval number (44-105). All samples were transported in sterile tubes containing 5 mL of nutrient broth (NB; Thermo Scientific Oxoid, Basingstoke, UK) to the Microbiology Laboratory in the Department of Biology, Faculty of Science, Taif University (Female Section). The bacterial swabs were cultured on nutrient agar (NA; Thermo Scientific Oxoid) for 24 h at 37 °C under aerobic conditions. Suspected *Bacillus* sp. colonies were identified based on morphological assessment, Gram staining, and biochemical tests, including catalase, oxidase, and motility, following standard protocols [36]. The morphological characteristics of the selected isolates were further examined using scanning electron microscopy (SEM).

### 2.3. Amplification and Sequence Analysis

Genomic DNA was used as a template, and bacterial universal primers 27F (5′-gagtttgatcactggctcag-3′) and 1492R (5′-tacggctaccttgttacactt-3′) were employed to amplify a partial sequence of the 16S rDNA gene by polymerase chain reaction (PCR). The PCR products were purified using DNA purification kits, and the purified DNA was sent to Macrogen Co. in Seoul, Korea for sequencing with the 518F and 800R sequencing primers. Sequence homology was assessed using Basic Local Alignment Search Tool (BLAST) to compare the obtained sequences with archived 16S rDNA sequences in GenBank (www.ncbi.nlm.nih.gov/nucleotide). Sequence data were collected, and a consensus sequence was generated using the BioEdit sequence alignment editor. ClustalX (1.83) was used for multiple sequence alignments [37]. Phylogenetic trees were generated using the neighbor-joining DNA distance method in MEGA 7 [38]. Tree topologies were assessed using bootstrap analysis with 1000 resampled datasets [39]. The partial 16S rRNA gene sequences of the selected eight *Bacillus* strains have been deposited in NCBI GenBank with assigned accession numbers.

### 2.4. Antibacterial and Synergistic Activity of ZnO-NPs

The antibacterial properties of the ZnO-NPs, CM, and their combination (1:1) were assessed using the resazurin microtiter dilution method [40,41]. *Bacillus subtilis* ATCC 6633 was used as a reference strain for antibiotic susceptibility testing [42]. Serial two-fold dilutions of the tested substances were prepared, ranging from 0.25 µg/mL to 128 µg/mL. A *Bacillus* sp. (1.5 × 10⁸ CFU/mL, 100 µL) was introduced into each well of 96-well microtiter plates (MTP; Greiner BioOne, Frickenhausen, Mannheim, Germany) containing 200 µL of Mueller–Hinton Broth (MHB) with varying concentrations of the tested materials. The plates were incubated for 18 h at 37 °C, followed by the addition of 20 µL of resazurin dye (0.1% *w*/*v* in distilled H_2_O). After an additional hour at 37 °C, the color change from blue/purple to pink indicated active cellular metabolism, whereas the presence of dark blue signified no bacterial growth. A Microplate Reader (Biorad, Hercules, CA, USA) measured microbial growth or inhibition at 600 nm. The minimal inhibitory concentration (MIC) was defined as the lowest concentration of a substance that prevented bacterial growth, indicated by blue wells [43]. All materials were tested in triplicate on the MTP. Following MIC determination for the ZnO-NPs, CM, and the ZnO-NPs-CM combination, 50 µL aliquots from each tube showing no visible bacterial growth were inoculated onto Mueller–Hinton Agar (MHA) and incubated at 37 °C for 24 h. The minimum bactericidal concentration (MBC) was defined as the concentration at which 99.9% of the bacterial population was killed, which was confirmed by the absence of bacterial growth on the agar plates after incubation [43].

The in vitro antibacterial interaction was assessed using the checkerboard synergy method based on the fractional inhibitory concentration (FIC) index, as described by Khalil et al. [40]. The ZnO-NPs and CM combinations were tested using this approach [44]. Two-dimensional checkerboard titrations were conducted using microdilution broth, with the ZnO-NP concentrations ranging from 1 µg/mL to 64 µg/mL and the CM concentrations ranging from 2 µg/mL to 128 µg/mL, applied in both horizontal and vertical orientations. The positive control included MHB and the bacterial solution, while the negative control contained only MHB. The MIC values of the antibacterial agents were determined by comparing the bacterial growth in the test wells with that in the positive and negative controls. The optical density (OD) of the microplate was measured at 600 nm before and after incubation. Each bacterial strain was tested in triplicate across three independent days.

### 2.5. FIC Calculation

The FIC was calculated to assess the interaction between the ZnO-NPs and CM according to Equation (1):

FIC index = FIC_ab_ + FIC_np_ (1)

where ab is the antibiotic; np is the nanoparticles; FIC_ab_ = (MIC of ab with np)/(MIC of ab alone); and FIC_np_ = (MIC of np with ab)/(MIC of np alone).

The FIC values were interpreted [45] according to the following ranges: FIC < 0.5, synergistic interaction; FIC > 4.0, antagonistic interaction; 0.5 ≤ FIC ≤ 1.0, additive effect; and 1.0 < FIC ≤ 4.0, indifference.

### 2.6. Cytotoxicity

#### 2.6.1. Cell Culture

Human tongue squamous carcinoma (CAL27) and normal fibroblast (HFB4) cells were cultured in flasks with 10% fetal bovine serum (FBS) and 50 mg/mL gentamicin in Dulbecco’s Modified Eagle’s Medium (DMEM). The cultures were maintained in a humidified incubator at 37 °C with 5% CO_2_. Daily observations were made using an inverted microscope. When cell growth reached 70–80% confluence, the cells were dissociated using trypsin [22].

#### 2.6.2. Cytotoxicity of ZnO-NPs

The cytotoxicity of the ZnO-NPs was evaluated using the MTT colorimetric assay, following the method described by Chen et al. [45]. The ZnO-NPs were introduced into 96-well tissue culture plates that contained either CAL27 or HFB4 cells. The quantities of the ZnO-NPs ranged from 0.25 µg/mL to 128 µg/mL. The plates were incubated at 37 °C for 24 h. After incubation, 0.02 mL of the MTT solution (5 mg/mL in PBS) was added to each well, followed by further incubation for 4 h at 37 °C with 5% CO_2_. Afterward, 150 μL of dimethyl sulfoxide (DMSO) was added to each well to dissolve the formazan crystals formed from MTT reduction. The absorbance at 490 nm was measured using a BIORAD microplate ELISA reader. Untreated cells were used as the positive control. Cell viability was calculated using the following formula. The half inhibitory concentration (IC_50_) of the ZnO-NPs was determined using GraphPad Prism 9.0 (GraphPad Software Inc., La Jolla, CA, USA).

$$\mathrm{Cell}\;\mathrm{viability}\%=\frac{At-Abt}{Ac-Abc}\times 100$$

where the components of the equation are as follows:

At = Absorbance of the tested ZnO-NP concentration;

Abt = Absorbance of the blank well with the corresponding ZnO-NP concentration;

Ac = Absorbance of control cells;

Abc = Absorbance of the blank well with culture medium.

### 2.7. Biofilm Formation Assay

The biofilm-producing capacity (BPA) was assessed using a modified version of the MTP method described by Stepanovic et al. [46]. Each well of the MTP contained 100 µL of tryptone soy broth (TSB) and 100 µL of bacterial culture (1.5 × 10⁸ CFU/mL) grown overnight. Positive control wells were inoculated with *Bacillus subtilis* ATCC 6633, while negative control wells were seeded with *B. subtilis* ATCC 168. After 48 h of incubation at 37 °C, the wells were washed three times with 300 µL of PBS to remove any unattached cells. To stain the biomass, 250 µL of a 0.1% crystal violet solution (Difco Laboratories Inc., Detroit, MI, USA) was added and incubated for 30 min. The wells were then washed three times with PBS to remove excess dye. Then, 200 µL of 70% ethanol was added to each well to release the crystal violet from the biofilm. The plates were sealed with parafilm and allowed to stand at room temperature for 30 min. The absorbance of the resulting crystal violet solution was measured at 590 nm using a microplate reader. The biofilm-forming potential of the isolates was classified as non-adherent, weakly adherent, moderately adherent, or strongly adherent [47]:

OD_cut_ = OD_average_ of the negative control + (3 × SD of OD of negative control);

OD ≤ OD_cut_: non-adherent;

OD_cut_ < OD ≤ 2 × OD_cut_: weakly adherent;

2 × OD_cut_ < OD ≤ 4 × OD_cut_: moderately adherent;

OD > 4 × OD_cut_: strongly adherent.

### 2.8. Identification of Clindamycin Resistance and Biofilm Formation Genes

Genomic DNA from the selected *Bacillus* isolates was extracted using a DNeasy Tissue Kit (Qiagen, Hilden, Germany). A putative CM resistance gene was identified via PCR amplification using the erythromycin ribosome methylase (*erm*C) primer pair (Table 1) [48]. Additionally, the *tasA* and *sipW* coding regions (Table 1) were used to identify biofilm-forming genes [49]. The PCR reaction mixture contained template DNA, 0.5 µM of each primer, 1.25 U of Taq polymerase, 10 mM dNTPs, and 2 mM MgCl_2_. The PCR cycle parameters and primers are provided in Table 1. The PCR-amplified products were sequenced by Bionics (Seoul, Korea) using their proprietary service. The web-based BLAST algorithm was used to compare the sequences of the amplified fragments with gene sequences in the NCBI database.

### 2.9. Statistical Analysis

Data analysis was conducted using Minitab 19.2020.1 (Minitab Inc., Chicago, IL, USA) and GraphPad Prism 9.0. The bactericidal efficacy of antimicrobials was compared using one-way ANOVA. The mean and standard deviation of three replicates were calculated. Statistical significance was determined at a *p*-value of 0.05 or less.

## 3. Results and Discussion

**Figure 1:**
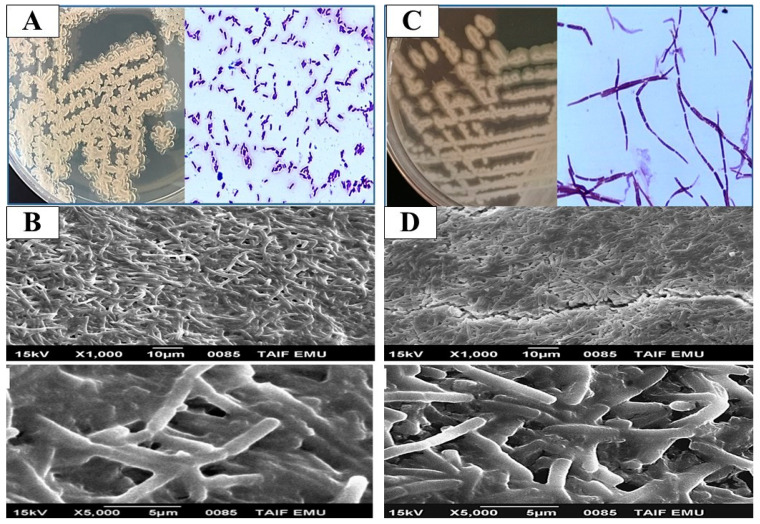
Cell morphology, Gram reaction, and scanning electron microscopy of representative isolates with robust biofilm-forming capabilities: *B. licheniformis* H1 (**A**,**B**) and *B. thuringiensis* H9 (**C**,**D**).

*Bacillus* species are part of the oral microbiota and play a key role in adapting to environmental changes within the protective biofilms that dominate the mucosal layer of the oral cavity [50,51]. While these microorganisms can persist as benign flora, they can also become opportunistic pathogens, leading to a variety of oral diseases. The microflora on the enamel are continuously adapting to environmental fluctuations within these biofilms [52]. Based on morphological and biochemical data, eight *Bacillus* isolates (16%) were identified from fifty isolates recovered from oral specimens. Our findings align with the morphological and biochemical characteristics typical of *Bacillus* species. The isolates were first confirmed at the genus level by assessing colony and cell morphology, catalase production, oxidase activity, motility, and Gram staining (Table 2). All isolates were identified as Gram positive, spore-forming, and motile rods (except the MD7 and MD8 strains, which were non-spore-forming and non-motile). All bacterial isolates tested positive for catalase production, except for the strain MD8. For the oxidase test, all isolates were oxidative, except for the H1 and H12 strains, which were characterized as non-oxidative bacteria (Table 2). To further investigate the biofilm-forming capabilities of representative strains, SEM was employed. Specifically, *Bacillus licheniformis* H1 and *Bacillus thuringiensis* H9 were examined (Figure 1). SEM images revealed that the bacterial cells were obstructed by extracellular secretions, and the extracellular matrix between the cells was also evaluated [3]. Several studies have demonstrated that oral cavity *Bacillus* species can be isolated [50,51,53].

**Figure 2:**
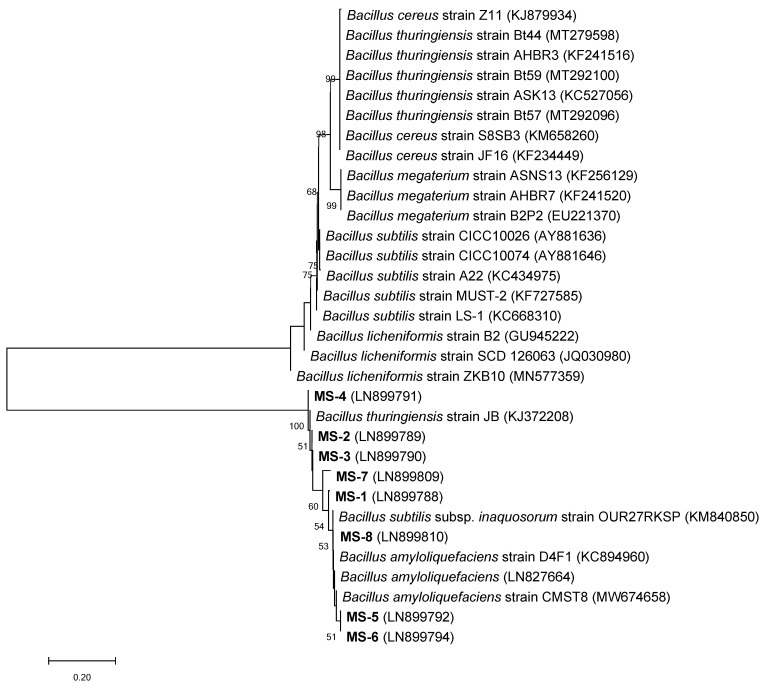
Neighbor-joining phylogenetic tree of the selected bacterial isolates (MS-1 to MS-8) based 16S rRNA. GeneBank accession numbers are given in parentheses. The scale bar (0.20) indicates a genetic distance.

To taxonomically identify the eight *Bacillus* species, the partial 16S rRNA gene sequence was used. Sequence homology was assessed using the BLAST tool on the NCBI website (http://www.ncbi.nlm.nih.gov/), comparing the 16S rRNA sequences with those of type strains available in the NCBI database (Table 3). Eight isolates were identified, MS-1, MS-2, MS-3, MS-4, MS-5, MS-6, MS-7, and MS-8, namely *Bacillus licheniformis* H1 (LN899788), *Bacillus cereus* H2 (LN899789), *Bacillus thuringiensis* H5 (LN899790), *Bacillus thuringiensis* H9 (LN899791), *Bacillus* subtilis H10 (LN899792), *Bacillus subtilis* H12 (LN899794), *Bacillus megaterium* MD7 (LN899809), and *Bacillus amyloliquefaciens* MD8 (LN899810), respectively. The phylogenetic relationship between the selected isolates and other related bacteria found in the GeneBank database is depicted in Figure 2.

Based on the MIC values of the ZnO-NPs, CM, and the combination of the ZnO-NPs and CM (Table 4) for each bacterial strain, their anti-biofilm formation activity was determined. As described above in the biofilm formation section, an estimate of biofilm formation was calculated. According to a microtiter dilution assay based on resazurin, all *Bacillus* strains were susceptible to the ZnO-NPs, with MIC values ranging from 1 to 64 µg/mL (Table 4). However, the CM-MIC values for the tested bacteria ranged from 2 to 128 µg/mL, with *Bacillus megaterium* MD7 showing the lowest MIC at 2 µg/mL. Compared to the ZnO-NPs or CM alone, the ZnO-NPs combined with CM exhibited superior antibacterial activity, with the MIC for the combination fluctuating between 0.25 and 16 µg/mL. This resulted in a 2–4-fold reduction in the MIC compared to the ZnO-NPs alone and a 4–8 fold reduction compared to CM alone (Table 4). The recombination of excitons in metal oxide nanoparticles may be hindered due to their small particle size and larger band gap. As a result, the increased availability of electrons leads to higher levels of reactive oxygen species (ROS), thereby enhancing the antibacterial properties of metal oxide nanoparticles [54]. The antibacterial efficiency of the ZnO-NPs was correlated with their ability to deform bacterial cells and induce ROS generation, as well as the release of Zn ions, leading to lipid peroxidation, protein oxidation, and DNA damage in bacterial cells [55]. The electrostatic interaction between the positively charged nanoparticles and negatively charged bacterial cells [56,57] was responsible for the suppression of bacterial growth and ROS production. The CM molecule contains reactive groups such as hydroxyl and amide that readily chelate with ZnO-NPs [55].

The interaction between CM and the ZnO-NPs resulted in synergy, as indicated by the FIC analysis of the ZnO-NPs-CM combination, shown in Table 5. The combination was synergistic for most *Bacillus* species tested, with FIC values of 0.375 for *B. licheniformis* H1 and *B. cereus* H2, 0.47 for *B. thuringiensis* H9, and 0.5 for *B. subtilis* H10, *B. megaterium* MD7, and *B. amyloliquefaciens* MD8. Only two strains, *B. thuringiensis* H9 and *B. subtilis* H12, exhibited additive interactions (Table 5). Due to its low MIC values, the solution containing the ZnO-NPs and CM showed a higher synergistic effect against the evaluated clinical isolates. By combining the ZnO-NPs with CM and other medications, we aim to mitigate the detrimental effects of antibiotics on the host, enhance their bactericidal efficacy, and reduce the spread of antibiotic-resistant bacteria [58,59].

**Figure 3:**
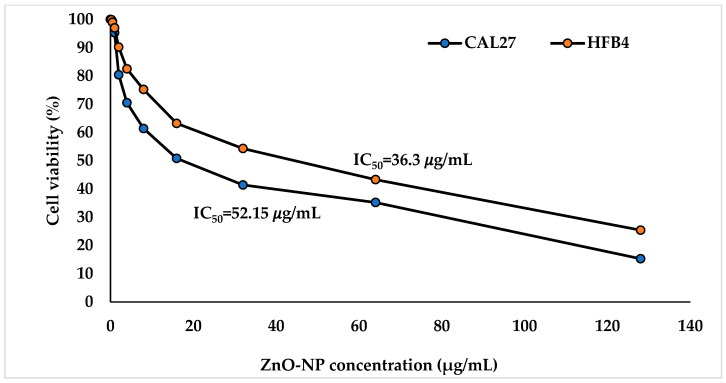
Cytotoxicity effect of ZnO-NPs on human tongue squamous carcinoma (CAL27) and normal fibroblasts (HFB4) cells. IC_50_: half inhibitory concentrations of ZnO-NPs.

The viability of cancer cells (CAL27) and normal fibroblast cells (HFB4) after treatment with varying doses of the ZnO-NPs is shown in Figure 3. The ZnO-NPs exhibited stronger cytotoxicity against CAL27 compared to HFB4. The viability of CAL27 and HFB4 cells after treatment with 0.25–128 µg/mL ZnO-NPs ranged from 99.8% to 15.3% and 99.9% to 25.4%, respectively (Figure 3). These findings indicate that the ZnO-NPs had an IC_50_ of 52.15 µg/mL for CAL27 cells and 36.3 µg/mL for HFB4 cells. This supports the reliability and selectivity of the ZnO-NPs, which is consistent with the findings of Babayevska et al. [57] and Naiel et al. [56], who demonstrated that the ZnO-NPs are more destructive to cancer cells than to normal cells at comparable concentrations. These results align with previous studies [59,60]. The cytotoxicity induced by the ZnO-NPs in cancer cells is linked to the generation of ROS. Chakraborti et al. [61] argued that ROS production is primarily responsible for the anticancer effects of PEG-modified ZnO-NPs.

A significant concern with *Bacillus* species is their resistance to many antimicrobials. The ability of these bacteria to form biofilms is a major factor contributing to their resistance to antimicrobial treatments, making bacterial eradication more challenging [46]. This study quantified the biofilm-forming capacity of *Bacillus* strains using the MTP test (Supplementary Figure S1). Notably, 62.5% of *Bacillus* strains were significant biofilm producers (Table 6). *B. licheniformis* H1, *B. thuringiensis* H9, *B. subtilis* H10, *B. megaterium* MD7, and *B. amyloliquefaciens* MD8 were identified as strong biofilm producers, while *B. cereus* H2 and *B. subtilis* H12 exhibited only modest biofilm production. The *B. thuringiensis* H5 strain produced less biofilm than the other strains. These results may help explain the higher resistance rates observed in *Bacillus* strains to CM in this study.

When combined with the expansion of germs that are resistant to antibiotics, the failure to create new medications has resulted in an increase in the number of deaths and illnesses, particularly in healthcare settings [62]. To address this issue, novel strategies for preventing and eradicating bacterial biofilms are urgently needed. This study comprehensively evaluated the use of nanoparticle–antibiotic combinations to reduce bacterial biofilms (Table 6). The combination of the ZnO-NPs and CM showed significant antibiofilm activity against most strains, reducing biofilm formation from strong or moderate to non-producing, except for *B. licheniformis* H1 and *B. thuringiensis* H9, whose biofilm production capacity became weak. In contrast, the ZnO-NPs or CM alone had minimal impact on the adherence of *Bacillus* strains to biofilms (Table 6). Previous studies have also investigated the antibacterial and antibiofilm properties of ZnO-NPs against *Bacillus* strains [63].

The antibacterial and antibiofilm activities of ZnO-NPs are believed to result from the generation of ROS, such as superoxide anions, hydroxyl radicals, and hydrogen peroxide, which are harmful to bacterial cells [64]. The release of Zn^2+^ ions, resulting from the accumulation of ZnO-NPs in the outer membrane of bacterial cells, leads to membrane disintegration, protein degradation, and genomic instability, ultimately causing bacterial cell death. ZnO-NPs can also readily interact with antibiotic compounds that contain active groups, such as hydroxyl and amide groups, through chelation, significantly increasing their antimicrobial effectiveness. This enhances the diffusion and permeation of ZnO-NPs through biofilms [65]. Based on the findings of Ryan et al. [66], it has been demonstrated that ZnO-NPs possess the capability to disrupt the efflux pump system, which is an essential component of bacterial resistance to numerous antibiotics. Furthermore, ZnO-NPs encourage the creation of free radicals, which have a strong interaction with thiol-containing proteins in the bacterial cell wall. This interaction leads to an increase in the rate at which bacterial cells are damaged [67].

Table 6 provides an overview of the antibiotic resistance and biofilm production profiles of the strains investigated in this study. Except for *B. thuringiensis* H5 and *B. subtilis* H12, the expression of the *ermC* antibiotic resistance gene was prominently observed in the *Bacillus* strains. All strains carried the *sipW* biofilm gene, except for *B. thuringiensis* H5. Two strains also contained the *tsaA* gene, which is associated with biofilm production (Table 7). These findings indicate that *B. licheniformis* H1 and *B. thuringiensis* H9 expressed genes involved in both biofilm formation and antibiotic resistance. Overall, a strong correlation was observed between the presence of *ermC* genes and the biofilm genotype in most *Bacillus* strains. Caro-Astorga et al. [49] demonstrated the roles of several genes, including *sipW* and *tasA*, in biofilm formation. The *tasA* gene is particularly important, as its product is associated with the formation of amyloid-like fibers, which are responsible for the floating biofilms of *Bacillus* species. The identification of *sipW* is critical, as it encodes a protease involved in the metabolism of *tasA*. Furthermore, many studies have shown that *erm* genes contribute to bacterial CM resistance [68]. Methylation of the ribosomal target sites for CM and erythromycin confers resistance to these antibiotics in *Bacillus* species [69]. Cross-resistance to erythromycin, CM, and streptogramin B is mediated by the macrolide–lincosamide–streptogramin B (MLSB) resistance mechanism [70]. This is the most common mechanism of resistance to macrolides and lincosamides, primarily mediated by *erm* genes [71].

## 4. Conclusions

The results of this study underscore the importance of *Bacillus* species in the oral cavity, demonstrating their potential in biofilm formation and antibiotic resistance, which complicate the treatment of oral and systemic infections. The molecular identification confirmed the presence of various *Bacillus* strains, each exhibiting distinct biofilm-producing and antibiotic resistance profiles. The synergy between the ZnO-NPs and CM significantly enhanced antibacterial activity and reduced biofilm production, suggesting that this combination could be an effective strategy for tackling biofilm-associated oral infections. These findings not only contribute to a better understanding of the antibiotic resistance mechanisms and biofilm formation in *Bacillus* species but also provide insights into novel approaches for oral infection management and systemic treatments by integrating metal oxide nanoparticles and antibiotic therapies. This holds great potential for advancing antimicrobial pharmaceuticals in the healthcare sector. In the near future, the dental care industry will benefit from enhanced insights into the modification of ZnO-NPs, which are promising antibacterial agents. However, future studies should explore the mechanisms of synergy between nanoparticles and antibiotics and evaluate their long-term efficacy and safety in clinical settings, particularly in biofilm-associated infections.

## Figures and Tables

**Table 1:** PCR cycle parameters and primers.

Gene(s)	Primer Sequence (5′ → 3′)	PCR Cycling Conditions
*ermC*	F-ATTGTGGATCGGGCAAATATT R-TGGAGGGGGAGAAAAATG	Initial denaturation of 5 min at 95 °C, followed by 30 cycles of 1 min at 95 °C, 30 s at 52 °C, and 1 min at 72 °C
*tasA*	F-AGC AGC TTT AGT TGG TGG AG R-GTA ACT TAT CGC CTT GGA ATTG	Initial denaturation of 5 min at 94 °C, followed by 40 cycles at 94 °C for 30 s, 59 °C for 45 s, 72 °C for 45 s, and final elongation at 72 °C for 5 min
*sipW*	F-AGA TAA TTA GCA ACG CGA TCTC R-AGA AAT AGC GGA ATA ACC AAGC	Initial denaturation of 5 min at 94 °C, followed by 40 cycles at 94 °C for 30 s, 54 °C for 45 s, 72 °C for 45 s, and a final elongation at 72 °C for 5 min

**Table 2:** Morphological and biochemical characteristics of the selected bacterial isolates.

Isolate Code	Gram Stain	Catalase	Oxidase	Motility	Sporulation	Cell Morphology	Colony Characteristics				
							Color	Form	Internal Surface	Margin	Elevation
MS-1	+	+	−	+	+	Short rod, single	Creamy with brownish center	Irregular	Glossy center, rough	Lobate	Umbonate
MS-2	+	+	+	+	+	Rods in pairs or chain	Brown	Irregular	Glossy	Entire	Slight raised
MS-3	+	+	+	+	+	Rods in chain	Creamy	Irregular	Glossy	Irregular	Raised
MS-4	+	+	+	+	+	Rods in long chain	Creamy	Irregular	Glossy	Irregular	Raised
MS-5	+	+	+	+	+	Short rod, single or pairs	Creamy	Irregular	Wrinkled	Undulate	Flat
MS-6	+	+	−	+	+	Short rod, pairs or chain	Creamy	Irregular	Wrinkled	Undulate	Flat
MS-7	+	+	+	−	−	Thick long rod, in chain	White	Circle	Glossy	Entire	Slight raised
MS-8	+	−	+	−	−	Thick short rod, single	Creamy	Irregular	Glossy	Entire	Flat

+, Positive result; −, negative result.

**Table 3:** Molecular identification based on BLAST comparison to the GeneBank database.

Isolate Code	Bacterial Species	Strain	Accession No.	Closest Relative (Accession No.)	Sequence Identity (%)
MS-1	*Bacillus licheniformis*	H1	LN899788	*Bacillus licheniformis* strain SCD 126063 (JQ030980)	99.50
MS-2	*Bacillus cereus*	H2	LN899789	*Bacillus cereus* strain JF16 (KF234449)	99.53
MS-3	*Bacillus thuringiensis*	H5	LN899790	*Bacillus thuringiensis* strain Bt57 (MT292096)	99.88
MS-4	*Bacillus thuringiensis*	H9	LN899791	*Bacillus thuringiensis* strain AHBR3 (KF241516)	99.24
MS-5	*Bacillus subtilis*	H10	LN899792	*Bacillus subtilis* strain CICC10026 (AY881636)	99.87
MS-6	*Bacillus subtilis*	H12	LN899794	*Bacillus subtilis* strain A22 (KC434975)	99.24
MS-7	*Bacillus megaterium*	MD7	LN899809	*Bacillus megaterium* strain ASNS13 (KF256129)	99.13
MS-8	*Bacillus amyloliquefaciens*	MD8	LN899810	*Bacillus amyloliquefaciens* strain D4F1 (KC894960)	98.94

**Table 4:** Antibacterial activity of ZnO-NPs-CM combination compared to CM and ZnO-NPs separately.

Strain	Tested Materials (µg/mL)					
	ZnO-NPs		CM		ZnO-NPs-CM Combination	
	MIC	MBC	MIC	MBC	MIC	MBC
*Bacillus licheniformis* H1	32	64	64	128	≤16	16
*Bacillus cereus* H2	4	8	8	16	1	2
*Bacillus thuringiensis* H5	64	64	≤128	≥128	≤16	16
*Bacillus thuringiensis* H9	16	32	32	64	≤8	≥8
*Bacillus subtilis* H10	≤32	32	≥32	64	8	≥16
*Bacillus subtilis* H12	8	16	16	32	≤2	4
*Bacillus megaterium* MD7	1	2	2	4	≤0.25	0.5
*Bacillus amyloliquefaciens* MD8	≤32	32	≥32	64	8	16

ZnO-NPs, zinc oxide nanoparticles; CM, clindamycin; MIC, minimum inhibitory concentration; MBC, minimum bactericidal concentration.

**Table 5:** FIC index of ZnO-NPs-CM combination against tested *Bacillus* strains.

Strain	* FIC Index	
*Bacillus licheniformis* H1	0.375	(S)
*Bacillus cereus* H2	0.375	(S)
*Bacillus thuringiensis* H5	0.75	(A)
*Bacillus thuringiensis* H9	0.45	(S)
*Bacillus subtilis* H10	0.5	(S)
*Bacillus subtilis* H12	0.75	(A)
*Bacillus megaterium* MD7	0.5	(S)
*Bacillus amyloliquefaciens* MD8M	0.5	(S)

S, synergy; A, additive; * FIC value interpretation: synergy ≤ 0.5, additive > 0.5 and ≤1.0.

**Table 6:** Efficiency of ZnO-NPs, CM, and their combination as anti-biofilm agents against selected strains.

Strain	Biofilm Formation							
	Without Treatment		ZnO-NPs		CM		ZnO-NPs-CM Combination	
	*A*_630 nm_	BPC	*A*_630 nm_	BPC	*A*_630 nm_	BPC	*A*_630 nm_	BPC
*B. licheniformis* H1	6.0 ± 0.5	S	2.0 ± 1.10	M	2.5 ± 1.02	M	1.0 ± 0.6	W
*B. cereus* H2	1.8 ± 1.2	M	1.0 ± 0.32	W	1.4 ± 0.61	W	0.38 ± 0.03	N
*B. thuringiensis* H5	1.0 ± 0.4	W	0.55 ± 0.11	N	0.36 ± 0.02	N	0.15 ± 0.02	N
*B. thuringiensis* H9	4.6 ± 1.32	S	1.8 ± 1.3	M	2.2 ± 0.5	M	1.0 ± 0.41	W
*B. subtilis* H10	3.9 ± 1.43	S	2.8 ± 1.0	M	2.0 ± 1.24	M	0.56 ± 0.00	N
*B. subtilis* H12	2.2 ± 0.45	M	2.0 ± 1.2	M	2.4 ± 0.71	M	1.8 ± 0.63	W
*B. megaterium* MD7	4.3 ± 1.20	S	1.9 ± 1.10	M	2.2 ± 1.14	M	0.46 ± 0.00	N
*B. amyloliquefaciens* MD8	4.5 ± 0.50	S	1.1 ± 0.10	W	2.4 ± 0.04	M	0.59 ± 0.00	N

BPC, biofilm-producing category; ZnO-NPs, zinc oxide nanoparticles; CM, clindamycin. Both antimicrobials (ZnO-NPs and CM) and their combinations were utilized at their minimum inhibitory concentrations.

**Table 7:** Antibiotic resistance and biofilm production profiles of the selected bacterial strains.

Bacterial Strains	Gene Detection		
	Biofilm Formation		Antibiotic Resistance
	* tasA *	* sipW *	*ermC*
*B. licheniformis* H1	+	+	+
*B. cereus* H2	−	+	+
*B. thuringiensis* H5	−	−	−
*B. thuringiensis* H9	+	+	+
*B. subtilis* H10	−	+	+
*B. subtilis* H12	−	+	−
*B. megaterium* MD7	−	+	+
*B. amyloliquefaciens* MD8	−	+	+

+; present, −; absent

## Data Availability

All data generated or analyzed during this study are included in this published article.

## Supplementary Materials

The following supporting information can be downloaded at https://www.mdpi.com/article/10.3390/pathogens14020138/s1, Supplementary Figure S1. MTP assay for detection of biofilm production.
