# Supplementary material for: Synergistic Antibacterial and Antibiofilm Effects of Clindamycin and Zinc Oxide Nanoparticles Against Pathogenic Oral Bacillus Species
Source: Pathogens. 2025 Feb 2;14(2):138. doi: 10.3390/pathogens14020138 (PMC11858533; doi:10.3390/pathogens14020138)
Supplement: Supplementary file 1 [file pathogens-14-00138-s001.zip › pathogens-3334473-supplementary.pdf]

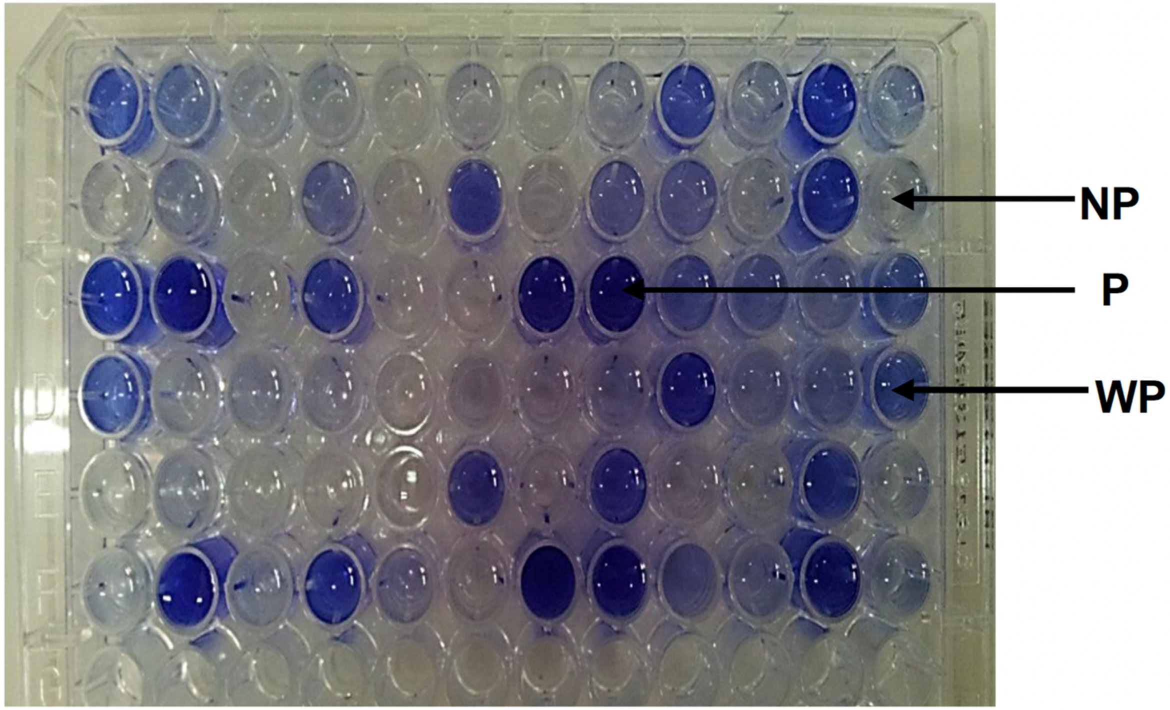

Supplementary Figure S1. MTP assay for detection of biofilm production. P: Producer > 0.240, WP: Weak Producer > 0.120 and < 0.240, NP: Non-biofilm Producer < 0.120. Positive results were biofilm producers (P) that appeared as deep blue color and weak biofilm producers (WP) that appeared as faint blue color. Negative results were non-biofilm producers (NP) that appeared as pale blue or colorless.
